# Subcutaneous Adipose Tissue in Female Volleyball Players: Is It Related with Performance Indices?

**DOI:** 10.3390/medicina56040159

**Published:** 2020-04-02

**Authors:** Sophia D. Papadopoulou, Amalia Zorzou, Antonio Garcia-de-Alcaraz, Thomas Rosemann, Beat Knechtle, Pantelis T. Nikolaidis

**Affiliations:** 1 Laboratory of Evaluation of Human Biological Performance, Department of Physical Education & Sport Science, Aristotle University of Thessaloniki, 57001 Thessaloniki, Greece; sophpapa@phed.auth.gr; 2 Exercise Physiology Laboratory, 18450 Nikaia, Greece; a.zorzou@hotmail.com (A.Z.); pademil@hotmail.com (P.T.N.); 3 Faculty of Educational Sciences, University of Almería, 04120 Almería, Spain; antoniogadealse@gmail.com; 4 LFE Research Group, Faculty of Physical Activity and Sport Sciences-INEF, Universidad Politécnica de Madrid, 28040 Madrid, Spain; 5 Institute of Primary Care, University of Zurich, 8091 Zurich, Switzerland; Thomas.Rosemann@usz.ch

**Keywords:** adiposity, age group, anthropometry, athletes, handgrip, playing position, vertical jump, women

## Abstract

*Background and objectives*: The aim of the present study was to examine subcutaneous adipose tissue distribution in female volleyball players with regards to (a) variation by anatomical site, (b) differences among age groups and playing positions, and (c) physiological characteristics associated with performance. *Materials and Methods*: Participants were adolescent (*n* = 89, age 15.6 ± 0.9 years, mean ± standard deviation) and adult female volleyball players (*n* = 78, 24.8 ± 5.3 years), who performed a series of anthropometric and performance tests including skinfold thickness in 10 sites, Abalakov jump (AJ) and handgrip test (HG). *Results*: Chin had the smallest thickness, and iliac crest and abdomen the largest. The largest correlations of skinfold thickness were shown with regards to AJ ad HG. Coefficient of variations in skinfold thickness correlated with performance indices with small magnitude. Triceps and chin were the most frequent predictors of performance indices. The anatomical site of skinfold was near the active muscle groups related to performance in HG. *Conclusions*: In conclusion, performance indices such AJ and HG were related with thickness of specific skinfolds and with the variation of thickness by anatomical site (i.e., the less the variation, the better the performance). Considering the relevance of specific skinfolds (e.g., triceps and chin) for performance, their further use would be recommended for purposes of training monitoring, volleyball players’ selection and talent identification.

## 1. Introduction

The relationship of fat mass and percentage of body fat (BF) with performance in volleyball has been identified since many decades [1,2], where it was observed that women of high performance level had low BF. Despite the changes in this sport since the 1970s, similar findings were shown recently. For instance, top elite volleyball players had low BF [3], volleyball players of the first national division had lower BF than those from the third division [4], and lower BF was found in those of higher ranked teams in the first division [5]. Moreover, lower BF was observed in volleyball players than non-athletes [6,7]. It has been also shown that BF varied by playing position in national level volleyball players with the largest score in setters [8]. In addition, BF has been found to correlate with performance-related measures such as vertical jump height, i.e., the lower the BF, the higher the jump height [7,9,10], and might change during preseason and competitive period [11]. It should be highlighted that the abovementioned studies considered the total fat mass and did not examine the role of fat distribution and particularly subcutaneous adipose tissue (SAT).

Fat distribution has been referred to the variation of fat across human body, which commonly was classified into arms, legs, and trunk [12,13,14]. It was observed that the national level volleyball players had lower BF than those of regional level, and both had lower BF and more even distribution of fat than non-athletes [12]. It has been also shown that during a four week preparatory period, total, trunk, arm, and leg fat decreased [13]. Fat distribution in the abovementioned studies on volleyball was evaluated using bio-impedance analysis (BIA).

Although these studies improved our understanding of regional fat in volleyball, several aspects remained less studied and the methodological approach of BIA had certain limitations (e.g., weak data resulting from this method, which can be severely misleading, particularly in lean athletes [15]). For instance, a potential use of skinfold thickness assessment method could provide details about more specific anatomical sites. Skinfold thickness referred to the sum of skin and SAT (including embedded fibrous structures) in an undefined compressed state (although the callipers used produce a standardized compression force). Moreover, the compression effect on the SAT thickness change is known to depend strongly on the measurement site (compressibility of SAT varies largely) and also on the investigated individual. It was acknowledged that other assessment methods of body composition (e.g., ultrasound technique) were more valid than skinfold thickness method, where it was supported that ultrasound technique provided an accuracy in the assessment of SAT not reached by any other method [16,17]. The ultrasound technique has the advantage of avoiding the tissue compression and movement that was shown in the use of skinfolds [18]. However, the ultrasound technique also had limitations in terms of cost of equipment, testing protocol, and preparation of participants that might explain the limited use of this method in female volleyball so far [17]. On the other hand, the skinfold thickness method was used widely by studies in female volleyball [19,20,21,22,23] and correlated very largely with BIA, air-displacement plethysmography method [24] and ultrasound technique [17].

Additionally, no information has been available about age- and playing position-related differences in SAT distribution. BF might vary by playing position, e.g., highest in liberos and lowest in centers [25], indicating an adaptation of body composition to specific demands of a playing role, and consequently, it was assumed that SAT distribution could differ among playing positions. In addition, female athletes at an age of 14–18 years were at a chronological period crucial for adherence to sport and further sport development [26]. A cut-off of 14 years was used previously to categorize female pubertal status into pre- and post-pubertal [27], and accordingly, female athletes of 14–18 years might be considered as post-pubertal, in a period of their life before adulthood and after having experienced large hormonal and growth changes. Therefore, the aim of the present study was to examine SAT distribution in female volleyball players with regards to (a) differences among anatomical sites, (b) differences among age groups and playing positions, and (c) physiological characteristics associated with performance. It was acknowledged that skinfold thickness referred to subcutaneous fat, and thus, the term “SAT distribution” was used to describe patterns of differences in skinfold thickness among anatomical sites for the purposes of this study. It was hypothesized that skinfold thickness would correlate more with physiological characteristics in anatomical sites close to the involved muscles than in those away from the muscle action, e.g., jumping performance would correlate more with lower limbs skinfolds than with skinfolds away from the main site of muscle action.

## 2. Materials and Methods

### 2.1. Study Design and Participants

A cross-sectional study design was used in the present research to analyze the variation of fat distribution by age, playing position, and performance indices. The data were collected during the competitive periods of seasons from 2010 to 2016 in our laboratory (EPL). A single dataset for each participant was considered during this period. All procedures adhered to the ethical guidelines of the Declaration of Helsinki 2013 and local institutional review board (EPL 2019/12). Participants were adolescent (*n* = 89, age range 14.0–18.0 years) and adult female volleyball players (*n* = 78, age range 18.2–41.7 years), who participated in age-specific official tournaments and the three highest national leagues (i.e., A1, A2 and B), respectively. They practiced volleyball three to four times per week, with each session lasting 90 min, and competed in an official match in the weekend. In addition, they were grouped as outside hitters (*n* = 58), centers (*n* = 40), opposites (*n* = 17), setters (*n* = 33), and liberos (*n* = 19) according to standard classification of playing position in this sport [25,28]. Prior to exercise testing, they were provided information about all procedures, and all participants (and their guardians in case of underage participants) provided their consent. Exercise testing was performed in an Exercise Physiology Laboratory by qualified personnel. Especially, with regards to the assessment of skinfold thickness, the personnel was trained during MSc and PhD studies with an emphasis to the particular 10 skinfolds of this study [29] and had an experience of using this assessment method in more than 10,000 subjects during the last 12 years. Each participant performed all the tests described below.

### 2.2. Equipment and Procedures

Participants were evaluated for stature (SECA, Leicester, UK) and body mass (HD-351 Tanita, IL, USA) to the nearest 0.1 cm and 0.1 kg, respectively. The thickness of 10 skinfolds (cheek, chin, pectoral, triceps, subscapular, abdomen, chest II, iliac crest, patella and proximal calf) was measured on the right side of the body to the nearest 0.1 mm (Harpenden, West Sussex, UK) [29]. Other popular approaches of skinfold thickness methods in volleyball used smaller number of anatomical sites, e.g., one (calf) [18], five skinfolds (triceps, biceps, subscapular, suprailiac, and calf [5]; triceps, subscapular, abdomen, suprailiac, and calf) [23] or seven (triceps, biceps, subscapular, supraspinale, abdomen, thigh, and calf) [20]. Although the majority of these studies used more than one skinfold, they focused more to the sum of skinfold thickness to estimate overall BF percentage rather than to examine patterns of SAT distribution among anatomical sites. The consideration of 10 skinfolds [29] in the present study allowed covering sufficiently human body and provided the opportunity to examine patterns of SAT distribution. Nevertheless, it should be noted that the SAT distribution referred to the comparison of the particular 10 skinfolds [29]; thus, other anatomical sites that also would be of relevance (e.g., lateral thigh, where women have large SAT depot) were not considered.

The skinfold method was selected to study SAT distribution in order for the findings to have practical relevance considering the widely cost-effective use of this method in field [5,19,20,23]. It was acknowledged that the DEXA method would not be an accurate method for SAT patterning analyses [30]. Fat masses of body parts obtained by DEXA were not comparable to tissue thicknesses obtained by skinfolds, and additionally, DEXA uses morphological assumptions that would not be applicable to highly trained athletes, which, in turn, might result in incorrect results such as negative BF values (e.g., lean athletes [30]). Ultrasound technique offering an accurate measurement of SAT could be an alternative option of choice. However, the ultrasound technique was not a standardized approach when the present study was started (2010) [16,18] and consequently, its use was not considered for our analysis.

The flexibility was assessed by the sit-and-reach test on a box providing 15 cm advantage [31]. Thereafter, participants cycled ergometer (828 Ergomedic, Monark, Sweden) with cadence 60 rpm for three steps each lasting three minutes against incremental braking force to estimate physical working capacity at heart rate (HR) 170 (PWC, W.kg^−1^) [32]. In addition, a three minute step test on a 30 cm step with cadence 24 ascents.min^−1^ evaluated exercise HR (recorded in the end of the test) [33]. Abalakov Jump (AJ) was performed on an Opto-jump (Microgate Engineering, Bolzano, Italy) platform [34]. Handgrip muscle strength (HG) was evaluated using a handgrip dynamometer (Takei, Tokyo, Japan) [35], the best trial of right and left hand was recorded and adjusted for body mass. Lastly, participants performed the Wingate anaerobic test (WAnT) on a cycle ergometer (874 Ergomedic, Monark, Sweden) against braking force 0.075 × body mass providing peak power (P_peak_, W.kg^−1^), mean power (P_mean_, W.kg^−1^) and fatigue index (FI, %) [36]. It should be considered—when interpreting the results of the WAnT—that this ergometer did not include the energy contained in the rotating flywheel [37]. These physiological characteristics were selected for this study considering their relevance either with health- or sport-related physical fitness. Considering the increased demands of female volleyball for anaerobic power and capacity, several studies used WAnT, single and multiple vertical jumps previously [38,39,40].

### 2.3. Statistical and Data Analysis

IBM SPSS v.23.0 (SPSS, Chicago, IL, USA) and Prism Graphpad v.7 were used for statistical analyses. Data were expressed as mean and standard deviation. Coefficient of variation (CV) of skinfolds thickness was calculated. A between-within subjects’ one-way analysis of variance (ANOVA) examined differences in thickness among the 10 skinfolds, the main effects of age group and playing position, and the age group × skinfold and playing position × skinfold interactions on thickness. Differences between age groups were examined using analysis of covariance with age as confounder. The magnitude of these differences was evaluated by eta square as small (0.01 ≤ η^2^ < 0.06), medium (0.06 ≤ η^2^ < 0.14), and large (η^2^ ≥ 0.14) [41]. An independent student t-test examined differences between adolescent and adult participants. The magnitude of these differences was evaluated by Cohen’s d, classified as trivial (d ≤ 0.2), small (0.2 < d ≤ 0.6), moderate (0.6 < d ≤ 1.2), large (1.2 < d ≤ 2.0), or very large (d > 2.0) [42]. The relationship of skinfolds thickness with performance indices was examined using Pearson correlation r and stepwise linear regression. The magnitude of Pearson r correlations was evaluated as, trivial (r ≤ 0.10), small (0.10 < r ≤ 0.30), moderate (0.30 < r ≤ 0.50), large (0.50 < r ≤ 0.70), very large (0.70 < r ≤ 0.90), and almost perfect (r > 0.90) [43]. Statistical significance was set at alpha 0.05.

## 3. Results

**Figure 1:**
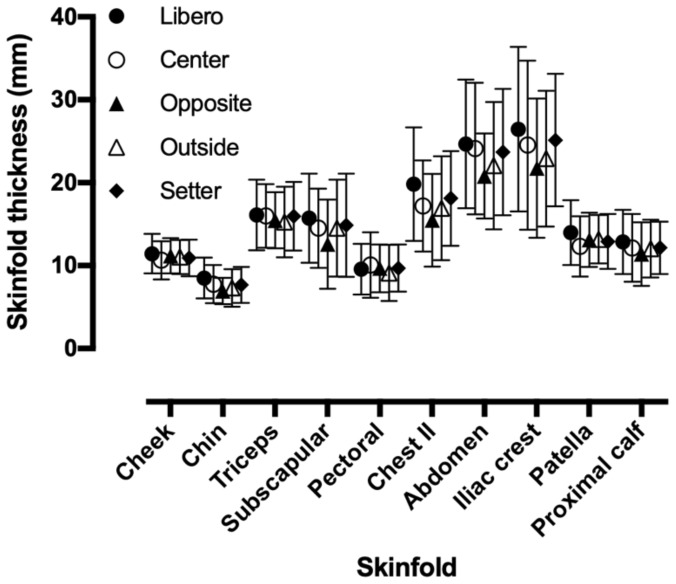
Variation of skinfold thickness by anatomical site and playing position.

The descriptive characteristics of participants can be seen in Table 1. A main effect of anatomical site on skinfold thickness was observed (*p* < 0.001, η_p_^2^ = 0.684), where chin (7.6 ± 2.2 mm) had the lowest score, and iliac crest (24.0 ± 8.9 mm) and abdomen (23.0 ± 7.5 mm) had the highest. No age group × anatomical site interaction on skinfold thickness was shown (*p* = 0.654, η_p_^2^ = 0.003). No difference between age groups was found in the other anatomical sites. No playing position × anatomical site interaction on skinfold thickness was shown (*p* = 0.413, η_p_^2^ = 0.025) (Figure 1). No difference in skinfold thickness among playing positions was observed for any anatomical site.

**Figure 2:**
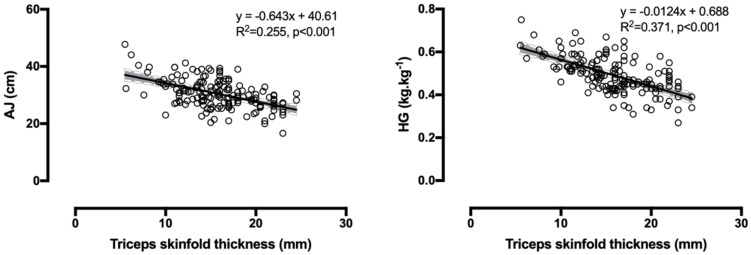
The relationship of Abalakov jump and handgrip muscle strength with skinfold thickness.

The correlations of skinfold thickness with PWC170, Step test, SAR and FI had trivial or small magnitude, with P_peak_ and P_mean_ were small or medium, with AJ were small, medium or large, and with HG were medium or large (Table 2). The strongest correlations were shown in Figure 2. All correlations between skinfold thickness and performance indices had the same direction, i.e., the larger the thickness, the lower the performance, which was presented by the negative values of r. The positive values of r in step test and FI were because high scores in these tests denoted low performance. In addition, CV correlated with Pmean (r = −0.25, *p* = 0.001), FI (r = 0.20, *p* = 0.013), AJ (r = −0.19, *p* = 0.015), and HG (r = −0.20 *p* = 0.010).

The results of the stepwise regression analysis can be seen in Table 3. Skinfold thickness had the highest prediction of performance for HG and AJ, and the lowest for FI and SAR. The most frequent predictors were triceps and chin, whereas five anatomical sites (cheek, pectoral, chest II, abdomen, and patella) were not predictors. Performance could be fully associated with skinfold thickness near the active muscle groups related to performance (HG), partially (AJ) or not (PWC170, step test, P_peak_, P_mean_, FI, SAR).

The correlation analysis of the total sample (Table 4) revealed small to very large relationship among skinfolds thickness. All correlations were in the same direction, i.e., the larger the one skinfold thickness, the larger the other one. Considering these correlations by age group, it was observed a trend of similar relationship among skinfolds thickness for both age groups.

## 4. Discussion

The main findings of the present study was that (a) the chin had the smallest thickness, and iliac crest and abdomen the largest; (b) no difference was observed in skinfold thickness between adolescent and adult participants; (c) neither age x anatomical site nor playing position × anatomical site interaction on skinfold thickness were observed; (d) the largest correlations of skinfold thickness were shown in AJ ad HG; (e) CV correlated with performance indices with small magnitude; (f) triceps and chin were the most frequent predictors of performance indices; and (g) the anatomical site of skinfold was near the active muscle groups related to performance in HG.

The SAT distribution in volleyball players (lowest score in chin, and highest in iliac crest and abdomen) reported in the present study was a novel finding. A study on abdominal, iliac crest, biceps, triceps, subscapular, calf and thigh skinfolds of non-athletes showed the highest score in thigh following by iliac crest [44]. Moreover, a research on triceps, subscapular, suprailiac, abdominal and thigh skinfolds found thigh as the largest one as well [45]. Furthermore, an examination of triceps, subscapular, biceps, iliac crest, suprailiac, abdominal, front thigh and medial calf showed front thigh as the largest one followed by abdominal [46]. Compared to these studies in non-athletes, participants had lower skinfold thickness in thigh (patella). Thus, it might be assumed that the long-term adaptations to volleyball training might explain the relatively low scores of leg skinfold thickness in the participants. Nevertheless, the role of the genetical background on SAT distribution should not be ignored as it has been shown that a part of the variance in SAT distribution might be accounted for by genetic factors [47]. Thus, SAT distribution in female volleyball players might be attributed to sport-specific training within the limits set by genetics.

Considering the frequency of skinfold thickness as predictor of performance indices, the findings were not surprising about triceps based on the literature on other sports. Triceps skinfold thickness was previously observed to discriminate elite from non-elite female softball players [48]. Furthermore, triceps skinfold was related with performance in female half-marathon runners [49]. It has been shown that race time in half-marathon women was moderately-to-largely correlated with pectoral, mid-axilla, triceps, subscapular, abdominal, suprailiac, and medial calf skinfolds [50].

With regards to the relationship between skinfold thickness and the proximity of its location with the locomotion needed for performance, a novel finding was that this relationship varied. For instance, it might be highlighted that triceps skinfold was relevant for HG. Although HG was considered a test of forearm muscle strength, the activation of arm muscles (e.g., biceps and triceps) would be necessary to stabilize elbow and forearm, where the forearm muscles originated. In addition, a performance index (AJ) was observed that related with both proximal (proximal calf) and distal skinfolds (triceps). An explanation of the role of triceps skinfold on AJ performance might be that—although the main site of muscle action was in the lower limbs—upper limbs also participated actively in the arm-swing movements preceding the take-off during this test. As was to be expected, skinfold thickness explained only a part of variance in performance indices. This finding was in agreement with previous research reporting negative relationship of these performance indices with body fat [10], i.e., the higher the BF, the lower the performance index. Although other correlates of performance were out of the scope of the present paper—apart from skinfold thickness—it was acknowledged that a large part of variance in performance indices was explained by variables such as muscle mass [40,51].

The lack of differences in skinfold thickness among playing positions or age groups might be attributed to the importance of body fat in this sport; although low values of body fat would be desired, volleyball players were not characterized by exceptional low body fat. The nutrition of participants was not examined; nevertheless, it was reasonable to assume that nutrition might account for a large variance of body fat of participants. For instance, it was observed that volleyball players had a diet of low caloric intake with low carbohydrates and high content of fat [52]. They consumed diet rich in fat [53,54] and an inadequate intake of vitamins was found even in high performance level [55]. It should be highlighted that a high fat diet did not imply decreased performance [56]. From a health perspective, considering the risk of under-weight athletes for female athlete triad, a concern of training and nutrition should be to achieve an optimal—instead of the lowest—BF [57]. For reference, competitive female volleyball players had BF ~21% to 25% [58,59].

A limitation of the present study was that it used skinfolds differing from those being widely used (e.g., patella instead of thigh or proximal calf instead of medial calf) and the standardized approach of ISAK; thus, caution would be needed to generalize the findings to different skinfolds considering the role of accurate site location [60] and to compare them with studies used ISAK approach. Another limitation was that factors such as biological age, age of onset of menarche and reproductive health were not considered. Although the role of these factors was recognized—especially with regards to the need for awareness of chronic low energy availability [61]—it was assumed that they would exert a lesser impact on our adolescent age group consisting of post-pubescents [27] (mean age 15.6 years) than in a potential sample of younger than 14 years. With this in mind, it was no surprise to observe no difference in anthropometric or physiological characteristics between adolescent and adult participants. Although the focus of our paper was on the role of SAT distribution on performance indices, it should be mentioned that volleyball performance was also depended on other anthropometric (body height) and physiological characteristics (muscle strength) [59,62].

On the other hand, this study was the first one to examine SAT distribution in volleyball players using skinfold thickness with regards to age, playing position and performance indices. Moreover, it should be highlighted that chin was not used widely as a skinfold; however, it correlated with several performance indices, and therefore, its inclusion in the anatomical sites used to evaluate skinfold thickness in female volleyball players should be encouraged. Considering the criticism about the relevance of skinfold thickness with total body fat [63], skinfold thickness might provide insight in SAT distribution even if this method would not be the most accurate one to estimate BF. Chin thickness correlated largely to very largely with skinfolds in torso with large fat stores (chest II, abdomen, and iliac crest), which might explain the relevance of chin skinfold for performance indices. Skinfold thickness was used widely in female volleyball, where it was shown that the number and anatomical sites of skinfolds varied by study [5,19,20,21]. Thus, the present study highlighted the importance of evaluating not only the sum of skinfolds, but also the patterns of SAT distribution among skinfolds. Moreover, the research hypothesis was verified partially, i.e., a relationship of performance index with skinfold thickness was observed in the case of handgrip muscle strength, but not in the other performance indices. Future studies should investigate further the relationship of performance indices with patterns of SAT distribution using other body composition assessment methods such as ultrasound to verify these findings.

## 5. Conclusions

In conclusion, performance indices such AJ and HG were related with thickness of specific skinfolds and with the variation of thickness by anatomical site (i.e., the less the variation, the better the performance). It was assumed that the underling physiological mechanism of these relationships was that these skinfolds reflected overall fat and were related with local muscle actions. Considering the relevance of specific skinfolds (e.g., triceps and chin) for performance, their further use was recommended for purposes of training monitoring, volleyball players’ selection and talent identification.

## Figures and Tables

**Table 1:** Descriptive characteristics (mean ± SD) of participants.

	Total (*n* = 167)	Adolescents (*n* = 89)	Adults (*n* = 78)	*p*	*η_p_^2^*
Anthropometric and physiological characteristics					
Age (years)	19.9 ± 5.9	15.6 ± 0.9	24.8 ± 5.3	-	-
Body mass (kg)	64.6 ± 9.2	63.3 ± 9.0	66.1 ± 9.1	0.386	0.005
Stature (m)	1.71 ± 0.07	1.69 ± 0.06	1.73 ± 0.07	0.364	0.005
AJ (cm)	30.5 ± 5.1	29.5 ± 4.9	31.6 ± 5.2	0.528	0.003
HG (kg.kg^−1^ of body mass)	0.49 ± 0.08	0.47 ± 0.08	0.52 ± 0.08	0.325	0.006
PWC (W.kg^−1^)	2.12 ± 0.55	1.96 ± 0.50	2.34 ± 0.54	0.880	<0.001
Step test (bpm)	153 ± 14	158 ± 13	148 ± 14	0.702	0.001
P_peak_ (W.kg^−1^)	8.88 ± 0.93	8.73 ± 0.81	9.04 ± 1.04	0.209	0.010
P_mean_ (W.kg^−1^)	6.54 ± 0.83	6.44 ± 0.76	6.67 ± 0.89	0.149	0.013
FI (%)	45.8 ± 8.4	45.4 ± 8.9	46.2 ± 7.8	0.517	0.003
SAR (cm)	25.4 ± 6.7	26.2 ± 6.1	24.4 ± 7.3	0.776	<0.001
Skinfold thickness					
Cheek (mm)	11.0 ± 2.2	11.6 ± 2.2	10.3 ± 2.0	0.090	0.017
Chin (mm)	7.6 ± 2.2	8.0 ± 2.2	7.2 ± 2.2	0.571	0.002
Triceps (mm)	15.7 ± 4.0	16.4 ± 3.6	14.9 ± 4.3	0.720	0.001
Subscapular (mm)	14.5 ± 5.6	15.1 ± 5.2	13.9 ± 5.9	0.966	<0.001
Pectoral (mm)	9.6 ± 3.3	9.6 ± 3.2	9.5 ± 3.5	0.656	0.001
Chest II (mm)	17.4 ± 6.0	17.8 ± 5.7	17.0 ± 6.3	0.321	0.006
Abdomen (mm)	23.0 ± 7.5	23.4 ± 7.4	22.7 ± 7.7	0.533	0.002
Iliac crest (mm)	24.0 ± 8.9	24.5 ± 8.6	23.4 ± 9.2	0.416	0.004
Patella (mm)	13.0 ± 3.3	13.4 ± 3.1	12.6 ± 3.6	0.807	<0.001
Proximal calf (mm)	12.1 ± 3.6	12.3 ± 3.4	11.93.9	0.683	0.001

SD = standard deviation, AJ = Abalakov jump, HG = handgrip muscle strength, PWC = physical working capacity, P_peak_ = peak power, P_mean_ = mean power, FI = fatigue index, SAR = sit-and-reach test. *P* value and eta square (η_p_^2^) referred to the results of one-way analysis of covariance.

**Table 2:** Correlations of performance indices with skinfold thickness.

Performance Index	Skinfolds									
	Cheek	Chin	Triceps	Subscapular	Pectoral	Chest II	Abdomen	Iliac Crest	Patella	Proximal Calf
AJ (cm)	−0.21 †	−0.38 ‡	−0.51 ‡	−0.38 ‡	−0.31 ‡	−0.35 ‡	−0.37 ‡	−0.40 ‡	−0.23 †	−0.45 ‡
HG (kg.kg^−1^)	−0.36 ‡	−0.42 ‡	−0.61 ‡	−0.41 ‡	−0.41 ‡	−0.39 ‡	−0.44 ‡	−0.45 ‡	−0.39 ‡	−0.39 ‡
PWC (W.kg^−1^)	−0.11	−0.23 †	−0.17 *	−0.22 †	−0.13	−0.18 *	−0.14	−0.20 *	0.02	−0.15
Step test (bpm)	0.08	0.24 †	0.22 †	0.19 *	0.14	0.13	0.13	0.21 *	0.09	0.19 *
P_peak_ (W.kg^−1^)	−0.22 †	−0.38 ‡	−0.42 ‡	−0.34 ‡	−0.33 ‡	−0.33 ‡	−0.35 ‡	−0.30 ‡	−0.12	−0.33 ‡
P_mean_ (W.kg^−1^)	−0.29 ‡	−0.40 ‡	−0.49 ‡	−0.42 ‡	−0.37 ‡	−0.42 ‡	−0.44 ‡	−0.42 ‡	−0.22 †	−0.38 ‡
FI (%)	0.18 *	0.05	0.12	0.11	0.12	0.12	0.16 *	0.21 †	0.16	0.09
SAR (cm)	−0.08	−0.21 †	−0.15 *	−0.17 *	−0.14	−0.19 *	−0.18 *	−0.14	0.02	−0.13

* *p* < 0.05, † *p* < 0.01, ‡ *p* < 0.001; AJ = Abalakov jump, HG = handgrip muscle strength, PWC = physical working capacity, P_peak_ = peak power, P_mean_ = mean power, FI = fatigue index, SAR = sit-and-reach test.

**Table 3:** Stepwise regression analysis.

Performance Index	Predictors	R	R^2^	SEE
AJ (cm)	Triceps, proximal calf	0.52	0.27	4.4
HG (kg.kg^−1^)	Triceps	0.61	0.37	0.07
PWC (W.kg^−1^)	Chin	0.23	0.05	0.54
Step test (bpm)	Chin	0.24	0.06	14.0
P_peak_ (W.kg^−1^)	Triceps, chin	0.45	0.20	0.84
P_mean_ (W.kg^−1^)	Triceps, subscapular	0.51	0.27	0.71
FI (%)	Iliac crest	0.21	0.05	8.2
SAR (cm)	Chin	0.21	0.04	6.6

SEE = standard error of the estimate. AJ = Abalakov jump, HG = handgrip muscle strength, PWC = physical working capacity, P_peak_ = peak power, P_mean_ = mean power, FI = fatigue index, SAR = sit-and-reach test.

**Table 4:** Correlations among skinfolds thickness.

Variable	Sample	Cheek	Chin	Triceps	Subscapular	Pectoral	Chest II	Abdomen	Iliac Crest	Patella	Proximal Calf
Cheek	Total	---									
	Adolescent	---									
	Adult	---									
Chin	Total	0.51 ‡	---								
	Adolescent	0.42 ‡	---								
	Adult	0.57 ‡	---								
Triceps	Total	0.52 ‡	0.64 ‡	---							
	Adolescent	0.40 ‡	0.64 ‡	---							
	Adult	0.60 ‡	0.61 ‡	---							
Subscapular	Total	0.36 ‡	0.66 ‡	0.58 ‡	---						
	Adolescent	0.29 †	0.66 ‡	0.56 ‡	---						
	Adult	0.40 ‡	0.66 ‡	0.58 ‡	---						
Pectoral	Total	0.35 ‡	0.53 ‡	0.59 ‡	0.53 ‡	---					
	Adolescent	0.22 *	0.41 ‡	0.54 ‡	0.52 ‡	---					
	Adult	0.52 ‡	0.67 ‡	0.64 ‡	0.54 ‡	---					
Chest II	Total	0.40 ‡	0.67 ‡	0.61 ‡	0.74 ‡	0.48 ‡	---				
	Adolescent	0.33 †	0.63 ‡	0.61 ‡	0.67 ‡	0.39 ‡	---				
	Adult	0.48 ‡	0.71 ‡	0.61 ‡	0.80 ‡	0.57 ‡	---				
Abdomen	Total	0.50 ‡	0.72 ‡	0.67 ‡	0.73 ‡	0.60 ‡	0.82	---			
	Adolescent	0.46 ‡	0.71 ‡	0.67 ‡	0.67 ‡	0.53 ‡	0.77	---			
	Adult	0.56 ‡	0.73 ‡	0.68 ‡	0.80 ‡	0.66 ‡	0.87	---			
Iliac crest	Total	0.49 ‡	0.65 ‡	0.64 ‡	0.68 ‡	0.59 ‡	0.80	0.86	---		
	Adolescent	0.49 ‡	0.59 ‡	0.63 ‡	0.57 ‡	0.51 ‡	0.73	0.82	---		
	Adult	0.51 ‡	0.71 ‡	0.66 ‡	0.78 ‡	0.66 ‡	0.87	0.89	---		
Patella	Total	0.47 ‡	0.32 ‡	0.49 ‡	0.20 †	0.37 ‡	0.34	0.36	0.42	---	
	Adolescent	0.36 †	0.21 *	0.43 ‡	0.20	0.34 †	0.39	0.43	0.55	---	
	Adult	0.58 ‡	0.40 ‡	0.52 ‡	0.18	0.41 ‡	0.30†	0.29 *	0.29 *	---	
Proximal calf	Total	0.34 ‡	0.49 ‡	0.67 ‡	0.41 ‡	0.55 ‡	0.46	0.55	0.46	0.55	---
	Adolescent	0.15	0.41 ‡	0.65 ‡	0.46 ‡	0.58 ‡	0.50	0.56	0.57	0.46	---
	Adult	0.55 ‡	0.57 ‡	0.69 ‡	0.37 †	0.53 ‡	0.42	0.53	0.59	0.53	---

*p < 0.05, † *p* < 0.01, ‡ *p* < 0.001.
